# Accuracy of ^18^F-flurodeoxyglucose-positron emission tomography/computed tomography in the staging of newly diagnosed nasopharyngeal carcinoma: a systematic review and meta-analysis

**DOI:** 10.2478/raon-2014-0011

**Published:** 2014-11-05

**Authors:** Balamurugan A. Vellayappan, Yu Yang Soon, Arul Earnest, Qing Zhang, Wee Yao Koh, Ivan Weng Keong Tham, Khai Mun Lee

**Affiliations:** 1 Department of Radiation Oncology, National University Cancer Institute, National University Health System, National University of Singapore, Singapore; 2 Centre for Quantitative Medicine, Office of Clinical Sciences, Duke-NUS Graduate Medical School, Singapore; 3 Department of Radiation Oncology, Sixth Hospital of Jiao Tong University, Shanghai, People’s Republic of China

**Keywords:** nasopharyngeal carcinoma, PET/CT, staging, accuracy, meta-analysis

## Abstract

**Background:**

The specific role of 18F-flurodeoxyglucose-positron emission tomography/computed tomography (FDG-PET/CT) in staging of nasopharyngeal carcinoma (NPC) remains to be validated. A systematic review and meta-analysis were performed to assess the accuracy of staging FDG-PET/CT for newly diagnosed NPC.

**Methods:**

We searched various biomedical databases and conference proceedings for relevant studies. We determined the pooled sensitivities and specificities, diagnostic odds ratios (DOR) and constructed summary receiver operating characteristic (SROC) curves using the hierarchical regression model.

**Results:**

15 relevant studies including 851 patients were identified. Five addressed primary tumor (T), nine addressed regional lymph nodes (N) and seven addressed distant metastasis (M). The combined sensitivity estimate for FDG-PET/CT in T classification was 0.77 (95% confidence interval [CI] 0.59–0.95). For N classification, combined sensitivity was 0.84 (95% CI 0.76–0.91), specificity was 0.90 (95% CI 0.83–0.97), DOR was 82.4 (23.2–292.6) and Q*-index was 0.90. For M classification, the combined sensitivity estimate was 0.87 (95% CI 0.74–1.00), specificity was 0.98 (95% CI 0.96–1.00), DOR was 120.9 (43.0–340.0) and Q*-index was 0.89.

**Conclusion:**

FDG-PET/CT showed good accuracy in N and M but not T classification for newly diagnosed NPC. FDG-PET/CT, together with Magnetic resonance imaging (MRI) of the nasopharynx, should be part of the routine staging investigations.

## Introduction

In 2008, there were approximately 84400 new cases of nasopharyngeal carcinoma (NPC) and 51600 deaths from the disease worldwide.^1^ The geographical disparities in the burden of NPC are noteworthy, with incidence rates highest in East and Southeast Asia and lowest in Central America.^1^

NPC may spread locally to involve the parapharyngeal soft tissue, base of skull or intracranial structures. The nasopharynx has a rich lymphatic plexus; 75% of patients present with enlarged cervical nodes, 80% of whom have bilateral involvement.^2^ NPC has a relatively high incidence of systemic metastasis (up to 41%) when compared with the other head and neck tumors (5%–24%). The most common sites of metastases are bone (20%), lung (13%), and liver (9%).^3^

NPC is staged non-surgically and treated primarily with radiotherapy (with or without chemotherapy). Accurate staging is essential as it influences the choice of treatment modalities, radiotherapy planning and prognosis. Combined modality treatment, as well as larger treatment volumes, invariably leads to greater toxicities. Although FDG-PET/CT is sometimes used in the clinical management of NPC in preference to other imaging modalities, such computed tomography or bone scans, the magnitude of benefit of using FDG-PET/CT, if any, is unclear. Indications for its use in the clinic have been rather empirical than standardized in many centres, often in the setting of a diagnostic dilemma affecting treatment options after the use of conventional imaging modalities.

The American Joint Committee on Cancer (AJCC) T (Primary tumor) N (Regional lymph nodes) M (Distant Metastasis) system is one of the most widely used staging system internationally.^4^ Conventional staging modalities may include MRI of the head and neck, contrast enhanced CT scans, liver ultrasound (US) and whole body radionuclide bone scan (WBBS). For M classification, one series reported the sensitivity and specificity for conventional workup (chest X-Ray, liver US, WBBS) to be 0.33 and 0.90 respectively; the same series reported CT of the thorax and abdomen with WBBS to be 0.67 and 0.92 respectively.^5^

The National Comprehensive Cancer Network (NCCN) guidelines recommend gadolinium-enhanced MRI of the nasopharynx and neck as well as CT scan (if indicated, for T and N classifications). It recommends imaging of distant metastases in the chest, liver and bones (which may include PET scan and/or CT) for patients with N2-3 disease. It also suggests that FDG-PET/CT scan may be considered for patients with Stage III and IV disease.^6^

The use of FDG-PET/CT has superseded stand-alone FDG-PET studies, by offering both functional and anatomic imaging, (for the initial staging and post-treatment assessments for a wide range of cancers).^7^ Published individual studies in the medical literature have reported increased accuracy especially in detection of metastases but are less conclusive on local and regional staging. The role of FDG-PET/CT in the overall staging of pre-treated NPC remains to be validated. To our knowledge, only one systematic review and meta-analysis of six studies examining the accuracy of FDG-PET/CT in detection of distant metastasis in pre-treated NPC showed it to have a high sensitivity of 88% and specificity of 97% for M classification.^8^ However, there were some limitations of this meta-analysis. Firstly, it did not address the accuracy of PET/CT scan for T and N classifications. Additionally, it excluded several publications in languages other than English^9^,^10^ and finally, new data^11^,^12^ have been published since the meta-analysis.

The aim of our study was to perform a systematic review and meta-analysis of all relevant publications to determine the accuracy of FDG-PET/CT in the TNM staging of newly diagnosed treatment naïve NPC patients, with reference to conventional modalities and/or clinical follow up.

## Materials and methods

### Identification and eligibility of relevant studies

We included studies, without language restriction, that determined the sensitivity and specificity of FDG-PET/CT for TNM staging of pre-treated (biopsy proven) nasopharyngeal cancer, when compared to conventional staging modalities (i.e. MRI or CT scan of head and neck for T and N classifications, biopsy or clinical follow up of suspected metastases to regional lymph nodes or distant sites).

We searched MEDLINE, Cochrane CENTRAL register of controlled trials, Cochrane Database of systematic reviews, Chinese national knowledge infrastructure (CNKI) and China Biomedical Literature Disc (CBMDisc) from date of inception to September 2011 and meeting proceedings of American Society for Radiation Oncology (ASTRO) and American Society of Clinical Oncology (ASCO) from 2000 to September 2011).

We used a search algorithm that included the following terms: (*1*) PET OR 18F-FDG PET OR positron emission tomography; (*2*) nasopharyngeal cancer OR nasopharyngeal carcinoma OR cancer of the nasopharynx OR lymphoepithelioma; (*3*) staging OR detection OR lymph node OR metastasis OR TNM.

FDG-PET only studies were excluded. For N and M classifications, studies that did not provide sufficient information to construct 2 × 2 table for sensitivity and specificity calculations were excluded. For T classification, we chose to analyze the sensitivity of FDG-PET/CT, in comparison to the reference standard. (*i.e* verifying false positive and true negative results in a non-surgically staged tumor would be impossible, and likely not reported in published studies).

The most recent publication was chosen when data was presented in more than one publication.

Two reviewers (B.V and S.Y.Y) independently judged study eligibility and disagreements were resolved by discussion and if necessary by a third reviewer (L.K.M)

### Data extraction

Two reviewers (B.V and S.Y.Y) extracted data from each eligible study independently using a standardized data extraction form and any disagreements were resolved by discussion or by appeal to a third reviewer (L.K.M).

Reviewers were not blinded with regard to information about the journal name, the authors, country of origin or the year of publication; as this has been shown to be unnecessary.^13^ In addition, we recorded the following information: study design (retrospective/prospective), sample size, age and gender distribution, stage of patients included and reference tests used to define extent of disease. Publications looking at more than one aspect of classification were treated independently. In particular, we extracted the number of cases that were true positive, false negative, true negative and false positive. True positive was defined as both FDG-PET/CT and the reference test detecting presence of disease; true negative where neither test detected disease; false positive where FDG-PET/CT detected disease but not the reference test and false negative where FDG-PET/CT failed to show disease detected by the reference test.

The methodological quality of each study was also independently assessed by B.V and S.Y.Y using the QUADAS tool.^14^ This scale contains 14 items that examine potential sources of bias in diagnostic studies in a systematic evidence-based manner. Higher scores are suggestive of lower risk of bias in the study’s methodology. Sensitivity analyses were performed after exclusion of retrospective studies, or studies with high risk of bias (QUADAS <10).

### Statistical analysis

The accuracy of FDG-PET/CT in the staging of newly diagnosed NPC was determined by the combined estimates of sensitivity and specificity, pooled diagnostic odds ratio (DOR), summary receiver operating characteristic (SROC) curves and Q*-index. The degree of heterogeneity among the included studies was assessed visually (forest plots) and statistically (chi-square tests and I2 statistic). When significant heterogeneity was observed (*P* <0.05), a random effects model was applied. A random effects meta-regression model was used to compare sub-group estimates.

The traditional ROC graph explores the effect of varying thresholds on sensitivity and specificity from a single study, unlike each data point in the SROC graph which represents a separate study. Thus, the SROC graph gives us a global estimate of the diagnostic test’s performance and illustrates the tradeoff between sensitivity and specificity.^15^ Q*-index is the best statistical summary method to reflect the diagnostic value. It is defined by the point where sensitivity and specificity are equal, which is the point closest to the ideal top-left corner of the SROC curve.^16^ The diagnostic odds ratio is a single indicator of test accuracy that combines data from sensitivity and specificity into a single number. It is the ratio of the odds of a positive test in a patient with disease relative to the odds of a positive test in a patient without disease and has a value that ranges from 0 to infinity, with higher values indicating better discriminatory test performance i.e. accuracy. A value of 1.0 indicates that the test does not discriminate between patients with and without the disease.^17^

Subgroups to be analyzed were determined a-priori, with the following reasons:

*T classification.* Contrast enhanced MRI is considered to the current gold standard for soft tissue involvement and intracranial extension.^18^ A subgroup analysis was performed considering studies, which utilized MRI to be the only acceptable reference test (versus MRI or CT or clinical findings). This may be viewed as a non-inferiority comparison or concordance of FDG-PET/CT to MRI.
*N classification.* FDG-PET/CT may over or underestimate the involvement of retropharyngeal and paraphayngeal lymph nodes; possibly because of poor distinction from the primary nasopharyngeal tumour.^11^ A subgroup analysis was done for studies looking primarily at cervical lymph nodal involvement versus non-cervical lymph nodes (*i.e*. retro/parapharyngeal). As neck dissection is not part of standard staging, it is unlikely to have histopathology as the reference standard. We performed a subgroup analysis to see if there was a difference between studies that required histology versus those that did not.
*M classification.* We performed a subgroup analysis to determine if there was a difference between studies which relied solely on clinical follow-up as the reference standard versus those which required histology.

Analyses were performed using META-DISC version 1.4 (XI Cochrane Colloquium; Barcelona, Spain)^16^ and STATA version 11.2 (Stata Corp, College Station, Tx, USA) and level of significance set at 5%.

## Results

### Study selection and description

**FIGURE 1.**
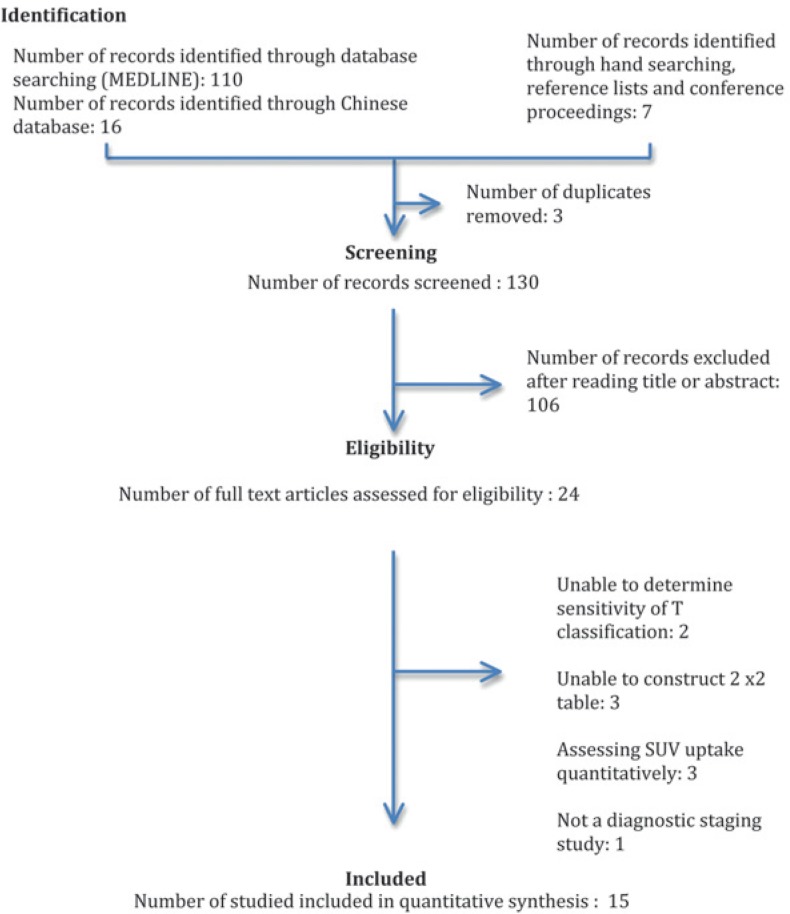
Flow of information through the different phases of a systematic review (as per PRISMA statement).

We identified 15 studies including 851 patients using the search strategy summarized in Figure 1. Five studies addressed the local extent of the primary tumor (T).^11^,^19^–^22^ Nine studies dealt with regional nodal classification, including retropharyngeal lymph nodal involvement (N).^10^,^11^,^19^,^20^,^23^–^27^ Seven studies dealt with distant metastatic classification (M).^5^,^9^–^12^,^19^, ^28^ One study was excluded from (M) as it potentially had overlapping data sets.^20^

Nine studies were published in the English language.^5^,^9^,^11^,^12^,^19^–^21^,^27^,^28^ One study was published as an abstract form.^10^ The characteristics of the 15 studies are summarized in Table 1. 220 patients were included in the analysis of T classification, 559 patients in N classification and 385 in M classification. The mean age of the participants was 46.8 years and approximately 70.5% were male. All studies except three included patients of all stages.^11^,^21^,^22^

Formal critical appraisal indicated that the methodological quality was high in three studies (QUADAS score ≥13),^11^,^20^,^26^ moderate in seven studies (QUADAS score 10–12)^5^,^11^,^19^,^22^,^23^,^25^,^27^ and low in five studies (QUADAS <10).^9^,^10^,^12^,^19^,^21^ Studies looking at more than one aspect of classification were assessed independently for quality. Most studies had a suboptimal design or insufficient description with regards to question 12 (100% no or unclear), question 11 (63% no or unclear) and question 4 (74% no or unclear).

All studies had a cross sectional design and ten of the 15 studies were conducted prospectively.^5^,^20^–^28^

### Accuracy

**FIGURE 2.**
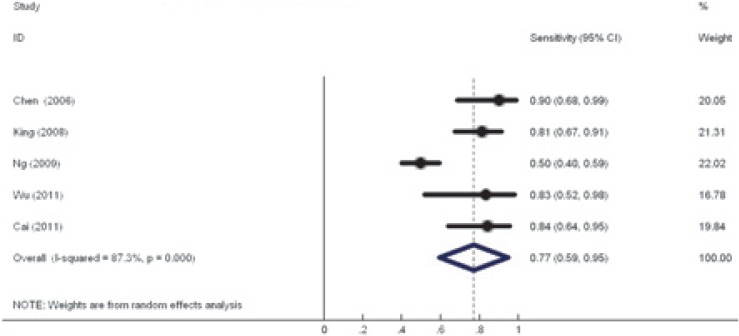
Pooled sensitivity for T classification.

*T classification.* Based on the combined data from five available studies that evaluated the T-classification our analysis revealed a sensitivity of 0.77(95% CI 0.59–0.95) while no specificity level could be ascertained (Figure 2). Four (of the five) studies did not report false positive results hence preventing us from calculating the specificity for T classification.^19^–^22^ Subgroup analysis revealed the sensitivity of FDG-PET/CT was lower when compared to MRI alone; however, this was not statistically significant (0.65 vs. 0.86, *P*=0.214). The sensitivity results on T classification were similar with exclusion of the two low quality studies,^19^,^21^ or the two retrospective studies.^11^,^19^

**FIGURE 3.**
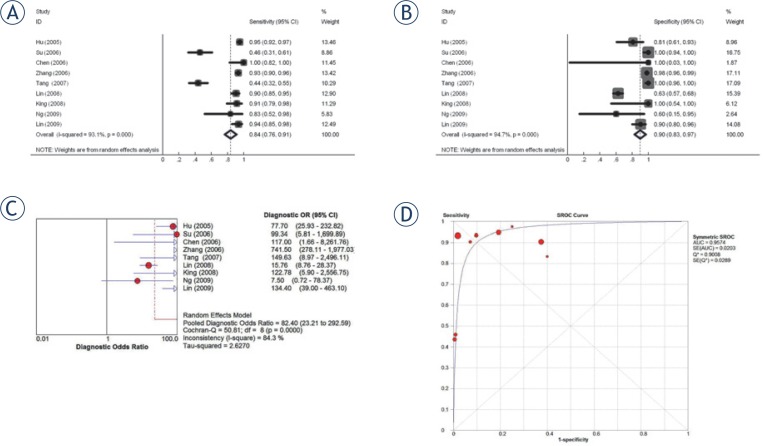
For N classification: **(A)** Pooled sensitivity **(B)** Pooled specificity **(C)** Pooled diagnostic odds ratio **(D)** Summary receiver operating characteristic (SROC) curve with Q*-index.

*N classification.* The combined sensitivity estimate for N classification is 0.84 (95% CI 0.76–0.91) and specificity 0.90 (95% CI 0.83–0.97). The pooled DOR for N classification was 82.4 (23.2–292.6). The Q*-index was 0.90 (SE 0.03) (Figure 3). The reference standards used for N classification varied amongst studies. MRI neck was the most frequently used reference standard.^11^,^24^,^26^,^27^ Two studies relied on clinical follow up to be their reference standard,^23^,^25^ and 2 other studies required histological confirmation though fine needle aspiration of involved cervical nodes.^10^,^20^ One study used contrast enhanced CT to be their reference standard^19^, which is considered to be inferior to MRI.^29^,^30^

The effect on sensitivity was significantly lower for studies assessing retro/parapharyngeal nodal involvement (0.94 vs. 0.44, *p*<0.001) whereas specificity did not differ significantly (0.85 vs. 1.00, *P*=0.305). There was no significant difference in sensitivity or specificity between studies that required histological confirmation (0.93 vs. 0.82, *P*=0.666; 0.82 vs. 0.91, *P*=0.533). The sensitivity and specificity results on N classification were similar with exclusion of two low quality studies,^10^,^19^ or the three retrospective studies.^10^,^11^,^19^

**FIGURE 4.**
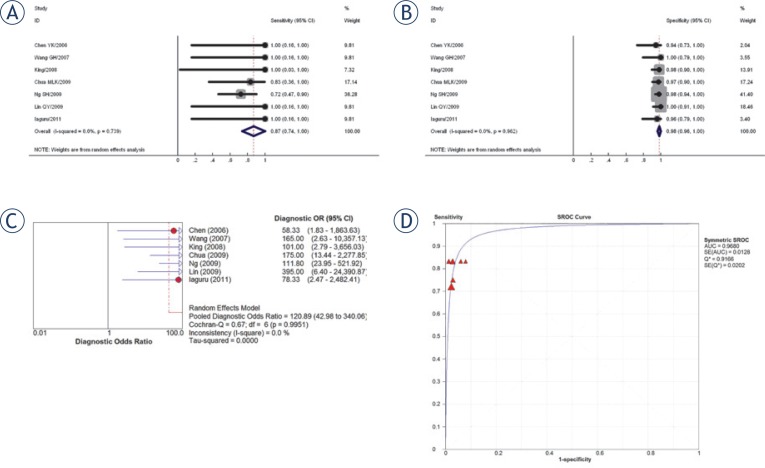
For M classification: **(A)** Pooled sensitivity **(B)** Pooled specificity **(C)** Pooled diagnostic odds ratio **(D)** Summary receiver operating characteristic (SROC) curve with Q*-index.

*M classification.* The combined sensitivity estimate for M classification is 0.87 (95% CI 0.74–1.00), and specificity 0.98 (95% CI 0.96–1.00). The pooled DOR for M classification is 120.9 (43.0–340.0). The Q*-index is 0.92 (SE 0.02) (Figure 4). All studies used either histological proof or clinical follow up (range 6–17 months) to define true positive and true negative lesions. Two studies used clinical follow up alone,^10^,^12^ and the duration was not reported. The mean time of follow up for the remaining studies was 12 months. Subgroup analysis did not show any significant differences for pooled sensitivity or specificity (1.00 vs. 0.84, *P*= 0.996; 0.99 vs. 0.98, *P*=0.531). Sensitivity analysis showed that the results on M classification were similar with exclusion of the three low quality studies,^9^,^10^,^12^ or the five retrospective studies.^9^–^12^,^19^

## Discussion

This meta-analysis suggests that FDG-PET/CT has excellent sensitivity and specificity compared to conventional staging modalities for N and M but not for T classification of NPC. We observed that FDG-PET/CT might be less accurate to determine involvement of para/retropharyngeal lymph nodes, although this estimate may be imprecise owing to relatively small number of studies.

Compared to other published meta-analyses investigating the accuracy of FDG-PET/CT, our results showed similar results for M classification but superior results for N classification. Nevertheless, we should note there are intrinsic differences. Kyazs *et al*. looked at the utility of FDG-PET (without combined CT) for cervical nodal metastasis in squamous cell head and neck cancer, referencing it against surgical specimens.^31^ The review did not find good evidence to support the routine use of pretreatment evaluation FDG PET. They reported an overall sensitivity and specificity of 0.79 and 0.86 respectively. The sensitivity was significantly lower in the clinically negative neck (0.50).

The variation in reported results may be due to the improved accuracy of integrated FDG-PET/CT versus stand-alone FDG-PET, differing reference standards (conventional methods versus surgical specimen) and differing primaries (NPC *versus* non-NPC). Our results did not differ after the inclusion of Chinese language publications for M classification, as previously reported by Xu and colleagues.^8^

The strengths of this study are that it addresses a pragmatic question, incorporates recently published data, includes Chinese language based publications, has a standardized study quality assessment, and has a pre-planned sub-group analysis to address potential sources of heterogeneity. Additionally, sensitivity analyses showed consistent results, suggesting the robustness of the findings.

There are some limitations of this meta-analysis. Firstly, our review was based on published results and not individual patient data. Secondly, the imaging reference standards used for T and N classifications were heterogeneous and subject to interpretation. The follow up time for M classification varied (6–17 months) and there was no consistent follow up strategy. Lastly, the included studies were heterogeneous in design though the majority of the studies were of low-moderate risk of bias based on the QUADAS assessment.

In conclusion, FDG-PET/CT showed good accuracy in N and M but not T classification for newly diagnosed pre-treated NPC. While head and neck MRI is still recommended for T classification, FDGPET/CT is accurate for clinical staging of regional nodes and distant disease and can be considered as an alternative standard of care wherever available. The diagnostic superiority of FDG-PET/CT over conventional staging modalities for detection of metastatic disease makes for more accurate disease prognostication and optimization of treatment strategy. The additional information derived from the FDG-PET/CT can also potentially aid neck nodal target delineation. FDG-PET/CT, together with MRI of the head and neck, has become part of the routine staging investigations for NPC at our centre. Future research should investigate the accuracy of FDG-PET/MRI as a single staging modality for NPC.^32^, ^33^

## Figures and Tables

**TABLE 1.** Characteristics of included studies

**Class.**	**Author (year)**	**Sample size**	**Design**	**Age (years)**	**Male (%)**	**Patient population**	**Reference Test**	**Quadas**
T	Chen (2006)^19^	20	R	46.3	70	All comers	nasoscopy and CT/MR	8
	King (2008)^11^	52	R	50	73	Stage III–IV	MRI	13
	Ng (2009)^20^	111	P	48.9	75.6	All comers	MRI	13
	Wu (2011)^21^	12	P	49	66.7	All comers, looking at intracranial and intraorbital extension (T4)	MRI/CT and clinical finding	9
	Cai (2011)^22^	25	P	50	64	Locally advanced NPC (at least T3)	MRI/CT and clinical findings	12
N	Hu (2005)^23^	105	P	43	78	All comers	Followup for 9 months	10
	Su (2006)^24^	53	P	40	68	All comers	MRI – looking at retropharyngeal LN	11
	Chen (2006)^19^	20	R	46.3	70	All comers	CT	8
	Zhang (2006)^25^	116	P	NR	79.3	All comers	Followup for 9 months	10
	Tang (2007)^26^	87	P	43	72.8	All comers	MRI - looking at para/retropharyngeal LN	13
	Lin (2008)^27^	68	P	41	58.5	All comers	MRI neck	11
	King (2008)^11^	52	R	50	73	Stage III–IV	MRI neck	12
	Ng (2009)^20^	17	P	48.9	75.6	All comers	FNA	13
	Lin (2009)^10^	41	R	NR	NR	All comers	FNA	7
M	Chen (2006)^19^	20	R	46.3	70	All comers	Histological proof, or clinical followup for 6 months	11
	Wang (2007)^9^	18	R	52	60.5	All comers	Histological proof, or clinical followup for 17 months (median)	9
	King (2008)^11^	52	R	50	73	Stage III–IV	Histological proof, or clinical followup for 12 months	12
	Chua (2009)^5^	78	P	50	76.9	All comers	Histological proof, or clinical followup for 6 months	11
	Ng (2009)^28^	150	P	48.1	74	All comers	Histological proof, or clinical followup for 12 months	11
	Lin (2009)^10^	41	R	NR	NR	All comers	Clinical followup (time not specified)	6
	Iaguru (2011)^12^	26	R	47.3	69.2	All comers	Clinical followup (time not specified)	9

FNA = fine needle aspirate cytology; M = distant metastasis; N = regional lymph nodes; NPC = nasopharyngeal carcinoma; NR = not reported; P = prospective; R = retrospective; T = primary tumor;

## References

[1] Jemal A, Bray F, Center MM, Ferlay J, Ward E, Forman D (2011). Global cancer statistics. CA Cancer J Clin.

[2] Chong VF, Ong CK (2008). Nasopharyngeal carcinoma. Eur J Radiol.

[3] Sham JS, Choy D, Choi PH (1990). Nasopharyngeal carcinoma: the significance of neck node involvement in relation to the pattern of distant failure. Br J Radiol.

[4] Edge SB, Byrd DR, Compton CC, Fritz AG, Greene FL, Trotti AM (2009). AJCC Cancer Staging Manual.

[5] Chua ML, Ong SC, Wee JT, Ng DC, Gao F, Tan TW (2009). Comparison of 4 modalities for distant metastasis staging in endemic nasopharyngeal carcinoma. Head Neck.

[6] NCCN guidelines for treatment of cancer by site. Head and neck cancers. http://www.nccn.org./professionals/physician_gls/f_guidelines.asp#head-and-neck.

[7] Lardinois D, Weder W, Hany TF, Kamel EM, Korom S, Seifert B (2003). Staging of non-small-cell lung cancer with integrated positron-emission tomography and computed tomography. N Engl J Med.

[8] Xu GZ, Guan DJ, He ZY (2011). (18)FDG-PET/CT for detecting distant metastases and second primary cancers in patients with head and neck cancer. A meta-analysis. Oral Oncol.

[9] Wang GH, Lau EW, Shakher R, Binns DS, Hogg A, Drummond E (2007). [Clinical application of (18)F-FDG PET/CT to staging and treatment effectiveness monitoring of nasopharyngeal carcinoma]. [Chinese]. Ai Zheng.

[10] Lin Q, Zhao H, Zhao J, Lin C (2009). Comparison of diagnostic value between 18F-FDG PET/CT and MRI in nasopharyngeal carcinoma. Journal of Jilin University.

[11] King AD, Ma BB, Yau YY, Zee B, Leung SF, Wong JK (2008). The impact of 18F-FDG PET/CT on assessment of nasopharyngeal carcinoma at diagnosis. Br J Radiol.

[12] Iagaru A, Mittra ES, Gambhir SS (2011). FDG-PET/CT in cancers of the head and neck: what is the definition of whole body scanning?. Mol Imaging Biol.

[13] Berlin JA (1997). Does blinding of readers affect the results of meta-analyses? University of Pennsylvania Meta-analysis Blinding Study Group. Lancet.

[14] Whiting P, Rutjes AW, Reitsma JB, Bossuyt PM, Kleijnen J (2003). The development of QUADAS: a tool for the quality assessment of studies of diagnostic accuracy included in systematic reviews. BMC Med Res Methodol.

[15] Moses LE, Shapiro D, Littenberg B (1993). Combining independent studies of a diagnostic test into a summary ROC curve: data-analytic approaches and some additional considerations. Stat Med.

[16] Zamora J, Abraira V, Muriel A, Khan K, Coomarasamy A (2006). Meta-DiSc: a software for meta-analysis of test accuracy data. BMC Med Res Methodol.

[17] Glas AS, Lijmer JG, Prins MH, Bonsel GJ, Bossuyt PM (2003). The diagnostic odds ratio: a single indicator of test performance. J Clin Epidemiol.

[18] Goh J, Lim K (2009). Imaging of nasopharyngeal carcinoma. Ann Acad Med Singapore.

[19] Chen YK, Su CT, Ding HJ, Chi KH, Liang JA, Shen YY (2006). Clinical usefulness of fused PET/CT compared with PET alone or CT alone in nasopharyngeal carcinoma patients. Anticancer Res.

[20] Ng SH, Chan SC, Yen TC, Chang JT, Liao CT, Ko SF (2009). Staging of untreated nasopharyngeal carcinoma with PET/CT: comparison with conventional imaging work-up. Eur J Nucl Med Mol Imaging.

[21] Wu HB, Wang QS, Wang MF, Zhen X, Zhou WL, Li HS (2011). Preliminary study of 11C-choline PET/CT for T staging of locally advanced nasopharyngeal carcinoma: comparison with 18F-FDG PET/CT. J Nucl Med.

[22] Cai L, Zhang W, Chen Y, Huang Z (2011). Value of ^18^F-FDG PET/CT and MRI for evaluating skull bone metastasis in nasopharyngeal cancer. Chongqing Medical Journal.

[23] Hu WH, Zhang GY, Liu LZ, Wu HB, Li L, Gao YH (2005). [Comparison between PET-CT and MRI in diagnosing nodal metastasis of nasopharyngeal carcinoma]. [Chinese]. Ai Zheng.

[24] Su Y, Zhao C, Xie CM, Lu LX, Sun Y, Han F (2006). [Evaluation of CT, MRI and PET-CT in detecting retropharyngeal lymph node metastasis in nasopharyngeal carcinoma]. [Chinese]. Ai Zheng.

[25] Tang LL, Ma J, Chen Y, Zong JF, Sun Y, Wang Y (2007). [The values of MRI, CT, and PET-CT in detecting retropharyngeal lymph node metastasis of nasopharyngeal carcinoma]. [Chinese]. Ai Zheng.

[26] Lin XP, Zhao C, Chen MY, Fan W, Zhang X, Zhi SF (2008). [Role of 18F-FDG PET/CT in diagnosis and staging of nasopharyngeal carcinoma]. [Chinese]. Ai Zheng.

[27] Ng SH, Chan SC, Yen TC, Chang JT, Liao CT, Ko SF (2009). Pretreatment evaluation of distant-site status in patients with nasopharyngeal carcinoma: accuracy of whole-body MRI at 3-Tesla and FDG-PET-CT. Eur Radiol.

[28] Olmi P, Fallai C, Colagrande S, Giannardi G (1995). Staging and follow-up of nasopharyngeal carcinoma: magnetic resonance imaging versus computerized tomography. Int J Radiat Oncol Biol Phys.

[29] Rumboldt Z, Gordon L, Bonsall R, Ackermann S (2006). Imaging in head and neck cancer. Curr Treat Options Oncol.

[30] Kyzas PA, Evangelou E, Denaxa-Kyza D, Ioannidis JP (2008). 18F-fluorodeoxyglucose positron emission tomography to evaluate cervical node metastases in patients with head and neck squamous cell carcinoma: a meta-analysis. J Natl Cancer Inst.

[31] Zaidi H, Del Guerra A (2011). An outlook on future design of hybrid PET/MRI systems. Med Phys.

[32] Loeffelbein DJ, Souvatzoglou M, Wankerl V, Martinez-Moller A, Dinges J, Schwaiger M (2012). PET-MRI Fusion in Head-and-Neck Oncology: Current Status and Implications for Hybrid PET/MRI. J Oral Maxillofac Surg.

